# Effect of a mindfulness-based cognitive behavior therapy intervention on occupational burnout among school teachers

**DOI:** 10.3389/fpsyt.2024.1496205

**Published:** 2025-01-24

**Authors:** Netra Raj Paudel, Prakash K. C., Clas-Håkan Nygård, Subas Neupane

**Affiliations:** ^1^ Unit of Health Sciences, Faculty of Social Sciences, Tampere University, Tampere, Finland; ^2^ Health and Population Department, Central Department of Education, Tribhuvan University, Kathmandu, Nepal; ^3^ Gerontology Research Center, Tampere University, Tampere, Finland

**Keywords:** burnout, intervention, mindfulness, CBT, educators, low-income countries

## Abstract

**Background:**

We aimed to assess the effect of mindfulness-based cognitive behavior therapy (MB-CBT) as an intervention to reduce burnout among secondary school teachers in Nepal.

**Methods:**

The baseline survey of this randomized controlled trial included 218 secondary school teachers from 37 schools in Kathmandu. At a one-month follow-up, 192 teachers completed post-test survey. The Maslach Burnout Inventory for Educators (MBI-ES) tool was used to measure burnout in three dimensions: emotional exhaustion (EE), depersonalization (DP), and personal accomplishment (PA). Teachers in the treatment arm (n=102) received MB-CBT, while those in the control arm (n=116) continued their regular activities. Linear regression models with generalized estimating equations were used to calculate the mean, mean change estimates, and their 95% confidence intervals (CIs).

**Results:**

The mean level of EE decreased during the post-test for both treatment (mean change -0.93, 95% CI: -2.56 to 0.70) and control (-1.71, 95% CI: -3.24 to -0.18) arms. The mean level of DP also decreased in the treatment arm (-0.12, 95% CI: -0.98 to -0.75), but a sharper decline was observed in the control arm (-1.97, 95% CI: -2.78 to -1.16). The mean level of PA increased in both the treatment (1.04, 95% CI: -0.21 to 2.30) and control (1.53, 95% CI: 0.35 to 2.70) arms.

**Conclusions:**

The prevalence of high EE and low PA decreased in both arms. However, the prevalence of high DP remained constant in the treatment arm but sharply decreased in the control arm. No significant differences between the arms were found in the post-test mean levels of EE, DP, and PA. The virtual MB-CBT intervention showed no significant impact on reducing burnout. Nonetheless, small reductions in burnout were observed in both groups.

## Introduction

Burnout is characterized by the gradual draining of emotional resources required to cope work-related stress (1, 2). Without proper management, burnout can evolve into a chronic condition, leading to physiological changes (3, 4). Over time, it can result in a steady decline in engagement, self-efficacy, and effectiveness, transitioning from enthusiasm to emptiness (5, 6). Moreover, burnout’s impact extends beyond the individual, negatively affecting the entire organization (7). Burnout diminishes employee motivation, reduces productivity, and fosters an unfavorable work environment marked by contagious negativity (8). The consequences are be severe, including higher absenteeism, reduced efficiency, and increased counterproductive behavior (9, 10).

Several factors contribute to burnout. Personality traits, particularly a Type A personality (11–13), and specific sociodemographic factors such as being young, single or unmarried, or a woman, increase susceptibility to burnout (14–16). Organizational factors such as low organizational commitment, job insecurity, excessive workload, role conflicts and ambiguities, limited support from supervisors and peers, and poor working conditions also contribute to burnout (17–19).

Given the demand and dedication required in their profession (20–22), burnout is reportedly more prevalent among educators (5, 6, 23, 24) than among other professionals. With the rapid increase in the prevalence of stress in recent years (25–27), the reported prevalence of perceived extreme stress (burnout) among educators has also risen (25, 28, 29). Nearly one-third of these stress levels may lead to clinical impairment (30). Therefore, to prevent further deterioration, it is necessary to identify early signs of stress and properly manage burnout among teachers (31). Burnout can be averted before it manifests and can also be effectively addressed in its early stages (21).

A recent scoping review (31) based on 40 articles published between 2018 and 2022 revealed that mindfulness interventions were associated reduced stress and burnout among educators. Similarly, our systematic review and meta-analysis (32) of 26 articles found that MB-CBT interventions effectively reduced teacher burnout. Hagermoser et al.’s (33) systematic review studied 18 articles published between 1987 to 2016 which identified meditation and mindfulness-based practices are the most commonly evaluated interventions for reducing teacher stress and burnout. Another study conclusively demonstrated that a brief mindfulness-based intervention effectively alleviated stress and burnout among teachers (34). Von der Embse et al.’s (35) systematic review which examined 24 articles published between 1998 and 2017, aligned with these findings and affirmed the effectiveness of mindfulness and cognitive behavioral interventions. These reviews collectively confirm that mindfulness-based CBT is an effective intervention for mitigating occupational stress and burnout among educators.

We aimed to test whether such interventions could also reduce burnout among community schoolteacher in the culturally and socio-economically different context of Nepal. The present study evaluated the effect of a mindfulness-based cognitive behavior therapy (MB-CBT) intervention on occupational burnout among school teachers in Nepal in a one month follow-up. We hypothesized that MB-CBT intervention would significantly reduce the occupational burnout among school teachers.

## Methods

The present study is a single-blind cluster randomized controlled trial, including two arms (treatment and control) to assess the impact of a four-week MB-CBT on burnout among school teachers in government-supported community schools in the Kathmandu district of Nepal.

The study protocol was registered in clinical trials (ClinicalTrials.gov ID: NCT05626543), and the ethical committee of Nepal Health Research Council (225/2022) granted ethical approval to conduct the study. Additional permission was obtained from the concerned schools and the Education Development and Coordination Unit in Kathmandu district.

### Study sample and randomization

**Figure 1:**
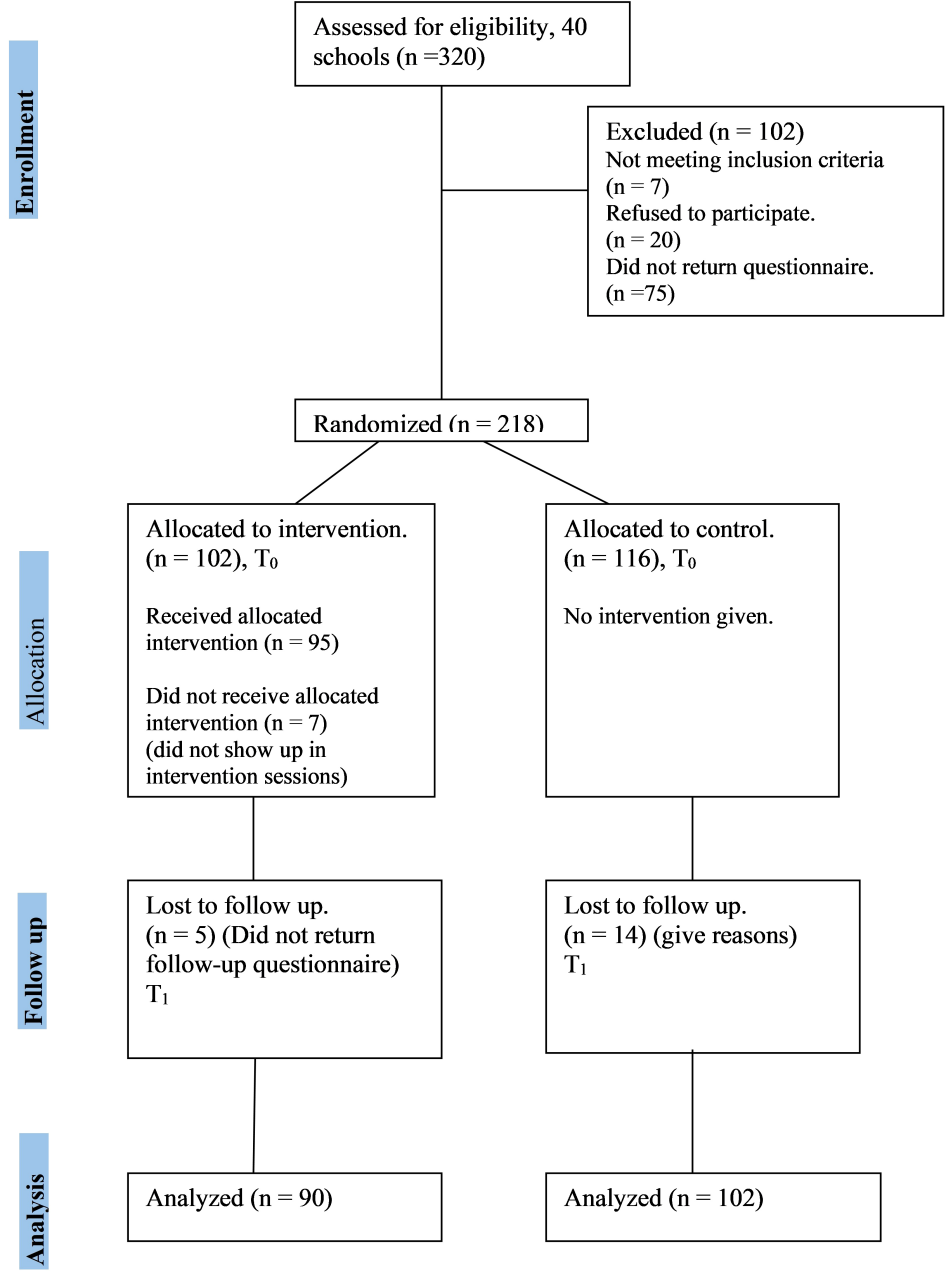
Flow diagram of the study.

The study population comprised secondary-level school teachers of community schools in the Kathmandu district of Nepal. We calculated the sample size proportional to the prevalence of occupational stress among school teachers. There was a need for a sample of 90 teachers in each study group and 180 in total and assuming a 10% attrition rate, the participants per study group was estimated at 100 and in total 200 teachers. Using a computer-generated random sampling method, we sampled 40 out of 165 eligible schools in the Kathmandu district. However, teachers from only 37 schools provided the consent. Altogether, 218 teachers replied to the baseline survey (**Figure 1**) out of the 291 who provided their consent, generating a response rate of 75% (36).

The web-based survey was conducted at baseline using a sociodemographic questionnaire to assess socio-demographics, lifestyles, health behaviors, work-related factors, and the Maslach Burnout Inventory for Educators (MBI-ES) tool for assessing occupational burnout. Participants at the school level were randomly divided into treatment (n=102) and control (n=116) arms considering balance by age and gender after the baseline survey. The treatment group received four weeks of virtual MB-CBT, while the control group continued regular activities.

A post-test survey was conducted one month after the intervention, in which 192 teachers (response rate 88%) responded to the questionnaire (102 teachers of the control arm and 90 teachers of the treatment). Teacher burnout was measured in the post-test using the same MBI-ES tool in the treatment and control arms.

### Outcome measurement tools

We used the MBI-ES tool, a validated and widely recognized instrument (37), to assess occupational burnout among teachers. The MBI-ES tool was obtained from Mind Garden with a license and translation agreement (MBI-ES Copyright ^©^1986 by Christina Maslach, Susan E. Jackson & Richard L. Schwab, California, USA). The MBI-ES tool comprised 22 items, which evaluate three distinct dimensions of occupational burnout: emotional exhaustion (EE), depersonalization (DP), and personal accomplishment (PA). Respondents were asked to rate their emotional states for each item on a 7-point Likert scale, spanning from “never” (0) to “every day” (6). The high burnout levels in each dimension were determined by the specific point score (38). Cronbach’s alpha coefficient (α) for the EE and DP subscales was 0.82 and 0.62, respectively, and the higher values in the subscale correspond to higher levels of burnout. On the other hand, the PA subscale, with eight items and a Cronbach’s alpha coefficient of 0.79, suggests lower levels of burnout with higher score (36).

### Measurement of other variables

The baseline measurement of socio-demographics, lifestyles, health behaviors, and work-related factors are described in detail elsewhere (36). Briefly, we measured demographic information on age, gender, ethnicity, income level, etc., as well as lifestyle factors such as smoking status, tobacco use, alcohol consumption, physical activity, self-rated health, physical fitness, sleep quality, comorbidity, and body mass index (BMI) using validated questionnaire tools. Similarly, work-related information such as ability to work, employment status, teaching hours per day, class size, and teaching students with special needs were measured at baseline.

### Intervention

**Figure 2:**
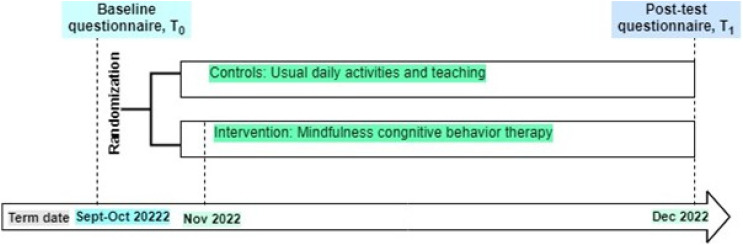
Timeline of participation in the study.

The principal investigator conceived and implemented an MB-CBT intervention for teachers of the treatment arm. The intervention manual was developed with two experts with over 20 years of experience. Drawing from contemporary literature on MB-CBT (39–43), the structured intervention was also facilitated by four expert professionals with over 15 years of experience in CBT. The program occurred between November 9 and December 11, 2022, for about a month and was held every four days with a total of eight sessions (**Figure 2**). The intervention sessions were conducted (44) via the online platform (Zoom) from 8 pm to 9 pm Nepal Standard Time (NPT), focusing on mindfulness-based cognitive practices (39, 42, 45) and providing in the Nepali language. Participant teachers were asked to practice one minute of loving-kindness meditation, five minutes of slow diaphragmatic breathing, five minutes of half-smile, five minutes of breath awareness, and ten minutes of body scan—twice daily on their own in the morning and evening. Flexibility was incorporated into the program, providing participants with recordings of previous sessions during the next session. The theoretical aspect was presented through PowerPoint slides and handouts, while practical sessions utilized audio-visual clips and pictures. Participants were assigned homework after each session, focusing on the upcoming session and reflecting on the immediate past session. Body scan audio instructions and keynotes were sent to participants via phone messages and social media. Two participants provided reflections after each session, and immediate, personal, and supportive counseling was offered to address any difficulties. The program was closely monitored by a second researcher who took notes, recorded the sessions, and responded to chat messages. On the contrary, the control group continued their regular activities; however, the intervention materials were provided to the teachers in the control arm after the follow-up survey.

### Statistical analysis

The study group first presented baseline characteristics of the studied population as frequencies and percentages. Linear regression models with generalized estimating equations (GEE) were used to assess changes in burnout (EE, DP, and PA) within and between the study groups from baseline to the post-test. The study groups were used as the between-subject factor, and time (baseline and post-test) as the within-subject factor. We calculated the mean, mean change, and 95% confidence intervals of all three dimensions of occupational burnout at baseline and post-test, and the difference between the study groups was tested. We conducted all the analyses considering intention-to-treat. Prevalence of occupational burnout in all three dimensions (high EE, high DP, and low PA) of burnout at baseline and post-test are also presented with their 95% CIs. We also calculated the interaction effect between the study group and time; as a sensitivity analysis, we presented the within and between-group difference in the mean level of burnout as mean change estimates and their 95% CI among those who replied at baseline and post-test.

All analyses were conducted using Stata statistical software (Release17. College Station, TX: StataCorp LLC).

## Results

**Table 1** shows the baseline characteristics of the study participants by study group. Of the 218 participants who replied to the baseline questionnaire, 116 were in the control, and the remaining 102 were in the treatment group. There was no statistically significant difference between the study groups in the baseline characteristics. Most of the participants were aged ≥40, male, married, had master’s degree qualifications, and had a monthly income exceeding 50,000 Nepali rupees. Most of the participants reported good self-rated health, no comorbidities, and had moderate to very good work ability. Among behavioral and lifestyle factors, most of the respondents had good sleep quality, were physically active, non-smokers, and non-alcohol drinkers. Most were teaching at least five hours/day and in classrooms with a higher number of students.

**Table 1:** Baseline characteristics of the studied population by study group.

Characteristics	*N=218*	Control (n=116)	Treatment (n=102)
Age			
< 40 years	85	44 (37.9)	41 (40.2)
≥ 40 years	133	72 (62.1)	61 (59.8)
Gender			
Female	72	37 (31.9)	35 (34.3)
Male	146	79 (68.1)	67 (65.7)
Marital status			
Married	198	106 (91.4)	92 (90.2)
All others	20	10 (8.6)	10 (9.8)
Education			
Bachelor’s	20	12 (10.3)	8 (7.8)
Master’s	188	101 (87.1)	87 (85.3)
MPhil or PhD	10	3 (2.6)	7 (6.9)
Income/month (NRs)			
≤50,000	96	52 (44.8)	44 (43.1)
>50,000	122	64 (55.2)	58 (56.9)
Teaching language			
Nepali	89	48 (41.4)	41 (40.2)
English	44	24 (20.7)	20 (19.6)
Both (Nep+Eng)	85	44 (37.9)	41 (40.2)
Employment status			
Permanent	129	65 (56.0)	64 (62.7)
Temporary	89	51 (44.0)	38 (37.3)
Self-rated health			
Good	146	82 (70.7)	64 (62.7)
Sub-optimal	72	34 (29.3)	38 (37.3)
Any disability			
No	196	104 (89.7)	92 (90.2)
Yes	22	12 (10.3)	10 (9.8)
Work ability			
Poor (0-6)	67	33 (28.4)	34 (33.3)
Moderate (7-8)	77	41 (35.3)	36 (35.3)
Very good (9-10)	74	42 (36.2)	32 (31.4)
BMI			
<25.0 kg/m^2^	119	59 (50.9)	60 (58.8)
≥25 kg/m^2^	99	57 (49.1)	42 (41.2)
Leisure time activity			
Sedentary	47	27 (23.3)	20 (19.6)
Spend with family	82	47 (40.5)	35 (34.3)
Social gathering	45	20 (617.2)	25 (24.5)
Kitchen/garden /Others	44	22 (19.0)	22 (21.6)
Physical fitness			
Good	146	82 (70.7)	64 (62.7)
Sub-optimal	72	34 (29.3)	38 (37.3)
Physical exercise			
Low	36	17 (14.7)	19 (18.6)
Moderate	75	42 (36.2)	33 (32.4)
High	107	57 (49.1)	50 (49.0)
Sleep quality			
Good	173	89 (76.7)	84 (82.4)
Poor	45	27 (23.3)	18 (17.6)
Smoking			
Never	191	99 (85.3)	92 (90.2)
Ever	27	17 (14.7)	10 (9.8)
Tobacco Chewing			
Never	197	102 (87.9)	95 (93.1)
Ever	21	14 (12.1)	7 (6.9)
Alcohol intake			
Never	156	81 (69.8)	75 (73.5)
Ever	62	35 (30.2)	27 (26.5)
Comorbidity			
No	163	85 (73.3)	78 (76.5)
one or more	55	31 (26.7)	24 (23.5)
Teaching hours/day			
≤4	73	45 (38.8)	28 (27.5)
5	114	56 (48.3)	58 (56.9)
≥6	31	15 (12.9)	16 (15.7)
Class size			
Normal	56	30 (25.9)	26 (25.5)
Overload	162	86 (74.1)	76 (74.5)
Employment Years			
<5	27	13 (11.2)	14 (13.7)
5-20	145	76 (65.5)	69 (67.6)
≥21	46	27 (23.3)	19 (18.6)
Teaching special need students			
Yes	34	18 (15.5)	16 (15.7)
No	184	98 (84.5)	86 (84.3)

**Figure 3:**
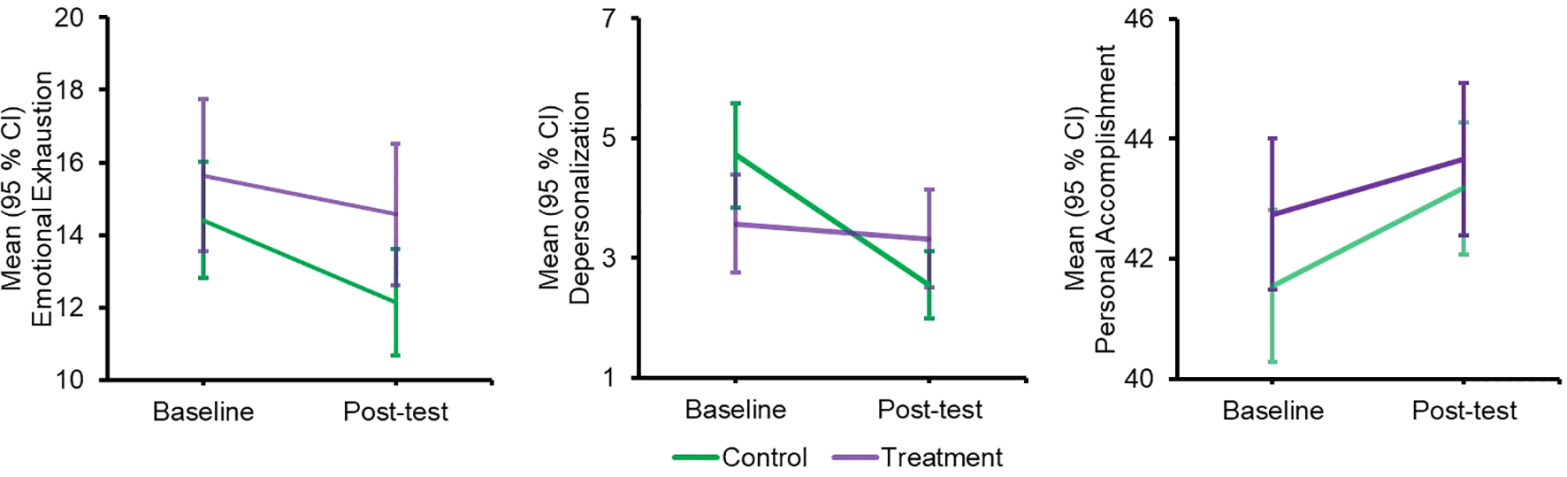
Mean (95% CI) emotional exhaustion, depersonalization and personal accomplishment during baseline and post-test.

**Figure 3** presents the mean with their 95% CIs of all three dimensions of occupational burnout of school teachers at baseline and post-test by the study groups. At baseline, the mean EE and PA in the treatment group were higher than the controls. At the post-test, the mean EE and DP decreased in the treatment group and controls. However, a sharper decrease was found in DP in the controls. The PA was increased within the treatment and the controls at the post-test. The mean scores for within-group change (**Table 2**) show a significant decrease in EE (-1.71, -3.24 to -0.18), DP (-1.97, -2.78 to -1.16), and an increase in PA (1.53, 0.35 to 2.70) in the control group. In the treatment group, decreased mean levels in EE (-0.93, 95% CI -2.56 to 0.70), and increased PA (1.04, -0.21 to 2.30) were found, which were not statistically significant while, decrease in DP (-0.12, -0.98 to -0.75) was statistically significant. Similarly, the changes between the study groups show an increased mean difference in EE and DP and decreased difference in PA from the baseline to the post-test. The interaction between the study group and time (baseline and post-test) was found to be significant for DP only.

**Table 2:** Mean difference in emotional exhaustion, depersonalization, and personal accomplishment during baseline and post-test.

MBI	Within Group (Post-test vs. Baseline)				Between Group (Treatment vs. Control)				*p*–value*
	Control		Treatment		Baseline		Post-test		
	Mean Difference	95% CI	Mean Difference	95% CI	Mean Difference	95% CI	Mean Difference	95% CI	
**Emotional exhaustion**	**-1.71**	**-3.24–** **-0.18**	-0.93	-2.56–0.70	1.23	-1.27–3.74	2.01	-0.59–4.62	0.49
**Depersonalization**	**-1.97**	**-2.78–** **-1.16**	-0.12	-0.98–0.75	**-1.15**	**-2.25–** **-0.04**	0.70	-0.46–1.87	0.002
**Personal accomplishment**	**1.53**	**0.35–2.70**	1.04	-0.21–2.30	1.20	-0. 51– 2.92	0.72	-1.08–2.51	0.58

*p–value for interaction between study group (control and treatment) and time (baseline and post-test), CI, Confidence Interval. The bold figures represent statistically significant result.

**Figure 4:**
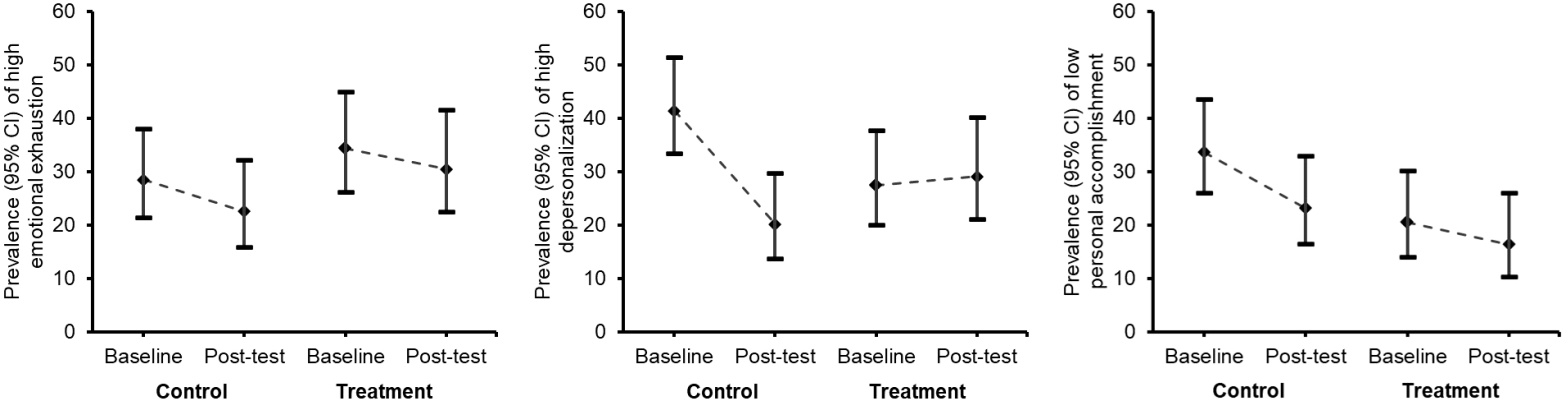
Prevalence (95% CI) of high emotional exhaustion, high depersonalization and low personal accomplishment during baseline and post-test.

**Figure 4** shows the change in the distribution of prevalence of occupational burnout (high EE, high DP, and low PA) with their 95% CIs among school teachers at baseline and post-test by study groups. The prevalence of high EE and low PA was decreased in both arms. However, the prevalence of high DP was constant in the treatment arm and sharply decreased in the control arm.

### Sensitivity analysis

We did additional analysis excluding the participants who were lost to follow-up at post-test. The results show a similar trend, except that DP for the treatment group had almost no change in the mean levels from baseline to the post-test (**Supplementary Table S1**). A similar trend was also found in the prevalence of high EE, high DP, and low PA (**Supplementary Table S2**).

## Discussion

This single-blinded cluster randomized controlled trial showed a modest positive impact of virtual MB-CBT on reducing occupational burnout among school teachers in Nepal; however, the reduction in occupational burnout was also observed among controls.

Baseline characteristics were comparable between the control and treatment groups, ensuring that observed post-test burnout differences could be attributed to the intervention rather than baseline disparities (46). The mean EE, DP and PA were comparable at baseline, with mean EE and PA were little higher in the control group. Post-test results showed a decrease in EE and DP in both groups, with a sharper decline in DP in the control group. PA increased in both groups, but not significantly. Overall, although not statistically significant we found positive changes in mean EE and PA, but this was also observed in the control group. A recent systematic review study shows that the effectiveness of mindfulness-based interventions may reduce certain dimensions of burnout, such as PA (47).

Overall, our findings indicate a positive effect of the MB-CBT intervention in reducing burnout among school teachers; however, the findings were not significant. We found a decrease in mean levels of burnout within the treatment arm. Many of the previous studies show the beneficial impact of MB-CBT in reducing burnout among teachers (31, 34, 48–55) and in other occupational groups (56–60). We used a virtual approach to deliver MB-CBT intervention for a month; a previous study reported this technique as effective (61). Our findings regarding the modest impact align with the findings of a meta-analysis of intervention studies, which suggested that mindfulness interventions lasting less than one month could have modest effects (62). We utilized a four-week MB-CBT for school teachers, targeting to reduce EE and DP and increase PA—all three dimensions of burnout (63).

Our findings contradict the generally expected outcome where the treatment group was anticipated to show a more significant improvement than the controls. Several reasons could explain this unexpected finding. First, the external factors or passage of time may lead to a natural reduction in burnout—even if it is not expected in a short time—across both groups. Second, the study participants may have been influenced by the awareness of being part of a study, leading to improved outcomes—the so-called Hawthorne effect (64, 65). Third, those in the control group may have realized that they were not part of the intervention group by the time the second survey was conducted, which was not explicitly communicated to them. However, it is possible that their awareness of not being part of the intervention could have influenced their response in the second survey. This may also explain the observed reduction in burnout within the control group, potentially due to compensatory rivalry or other related factors.

The major strength of this study is its design (two arms, single-blinded, cluster randomized), which establishes a strong internal validity framework (66), promoting confident causal inferences about the effects of the MB-CBT intervention. Digital surveys and virtual interventions were utilized to represent a cost-efficient and time-conserving approach (67, 68). The intervention was developed based on contemporary literature and the findings of a recent systematic review on the effectiveness of interventions in reducing stress and burnout among school teachers (32), strengthening its reliability and reproducibility. We used the Maslach Burnout Inventory (MBI-ES) tool, which is widely recognized for its robustness and accuracy in measuring occupational burnout (37), and the survey questionnaire was sent to both groups simultaneously avoiding chance of possible bias due to measurement instrument and timing of assessment. The study participants were balanced at baseline, and the sensitivity analysis contributed to the overall robustness of the findings.

However, the diverse backgrounds of participants and self-reported data pose challenges to eliminating bias compared to controlled laboratory clinical trials. Online delivery of MB-CBT may also raise concerns about participant engagement and consistency of intervention fidelity. The intervention was delivered quite late in the evening (8-9 pm) when most teachers are tired already which might also affect their willingness to accept and utilize the intervention properly. However, this was the only suitable time for most teachers especially the female teachers who also have lots of household responsibilities to be completed before they can join the intervention session. Generalizability may be limited to low-resource settings. The non-significant differences in the intervention in burnout dimensions suggest avenues for further exploration. More research is required to compare the effectiveness of MB-CBT interventions in virtual and physical formats. For future research, incorporating objective measurements, such as cortisol assessments besides self-reported data, is advisable. This dual approach can provide a more comprehensive understanding by combining subjective self-assessments with objective physiological measurements. The study’s focus on a specific group of teachers in Nepal may limit the generalizability of the findings to other similar populations or cultural contexts. The time constraints teachers face, and the challenges of adapting these programs to different contexts require careful consideration.

## Conclusions

In conclusion, the study indicates a moderate impact of virtual MB-CBT on reducing occupational burnout among school teachers in a one-month follow-up. The intervention had statistically no significant impact on occupational burnout. However, a reduction in burnout was found in both groups, with a sharper reduction in the control group, warranting further studies and explanation.

## Data Availability

Data from this study can be requested at netra.paudel@tuni.fi.
