# Supplementary material for: Effect of a mindfulness-based cognitive behavior therapy intervention on occupational burnout among school teachers
Source: Front Psychiatry. 2025 Jan 24;15:1496205. doi: 10.3389/fpsyt.2024.1496205 (PMC11802515; doi:10.3389/fpsyt.2024.1496205)
Supplement: Supplementary file 1 [file Table1.docx]

**Effect of a mindfulness-based cognitive behavior therapy intervention on occupational burnout among schoolteachers**

**Paudel et al. 2024**

**Supplementary results**

**Table S1:** Difference in mean scores and standard deviation (SD) of occupational burnouts of schoolteachers at pre and post-test by study group. The analysis includes only those who replied at baseline and at post-test (n=192)

| **MBI** | **Treatment** | **Control** | ***P*-vlaue** |
| --- | --- | --- | --- |
| **T_0_** |  |  |  |
| Emotional Exhaustion | 15.41 (10.72) | 13.53 (7.71) | 0.160 |
| Depersonalization | 3.30 (4.11) | 4.30 (4.36) | 0.103 |
| Personal Acomplishment | 42.52 (6.64) | 41.73 (7.02) | 0.427 |
| **T_1_** |  |  |  |
| Emotional Exhaustion | 14.57 (9.51) | 12.14 (7.62) | 0.051 |
| Depersonalization | 3.32 (4.00) | 2.55 (2.94) | 0.126 |
| Personal Acomplishment | 43.67 (6.18) | 43.18 (5.69) | 0.568 |

**Table S2:** Prevalence of low, moderate, and high occupational burnout among schoolteachers at pre- and post-test by study group. The analysis includes only those who replied at baseline and at post-test (n=192)

| **MBI Scoring** | **Treatment** | | | **Control** | | | **P value** |
| --- | --- | --- | --- | --- | --- | --- | --- |
|  | **Low** | **Moderate** | **High** | **Low** | **Moderate** | **High** |  |
| **T_0_** |  |  |  |  |  |  |  |
| Emotional Exhaustion | 60 (43.8) | 20 (47.6) | 10 (76.9) | 77 (56.2) | 22 (52.4) | 3 (23.1) | 0.073 |
| Depersonalization | 69 (76.7) | 16 (17.8) | 5 (5.6) | 62 (60.8) | 33 (32.4) | 7 (6.9) | 0.053 |
| Personal Acomplishment | 71 (78.9) | 9 (10.0) | 10 (11.1) | 71 (69.6) | 20 (19.6) | 11 (10.8) | 0.175 |
| **T_1_** |  |  |  |  |  |  |  |
| Emotional Exhaustion | 63 (70.0) | 18 (20.0) | 9 (10.0) | 81 (79.4) | 17 (16.7) | 4 (3.9) | 0.177 |
| Depersonalization | 65 (72.2) | 22 (24.4) | 3 (3.3) | 82 (80.4) | 19 (18.6) | 1 (1.0) | 0.294 |
| Personal Acomplishment | 75 (83.3) | 8 (8.9) | 7 (7.8) | 80 (78.4) | 15 (14.7) | 7 (6.9) | 0.461 |
